# Relations of Life’s Essential 8 score with arterial and microvascular function: The Jackson Heart Study

**DOI:** 10.1177/1358863X261444663

**Published:** 2026-06-23

**Authors:** Arko S Dhar, William B Hillegass, Leroy L Cooper, Abu Yusuf Ansari, Ramachandran S Vasan, Gary Mitchell, Ervin R Fox

**Affiliations:** 1 University of Mississippi Medical Center, Jackson, MS, USA; 2 Vassar College, Poughkeepsie, NY, USA; 3 Jackson Heart Study, Coordinating Center, Jackson, MS, USA; 4 University of Texas School of Public Health, San Antonio, TX, USA; 5 Cardiovascular Engineering, Inc., Needham, MA, USA

**Keywords:** cardiovascular disease, disease prevention, Life’s Essential 8 (LE8), risk factors, vascular complications, vascular physiologic testing

## Abstract

**Background:**

The American Heart Association (AHA) Life’s Essential 8 (LE8) provides a means of scoring cardiovascular health but has yet to be correlated with vascular function in an African American cohort.

**Methods:**

In a sample of Jackson Heart Study participants (*N* = 2186, mean age 57 years, 65% women), LE8 scores were calculated per AHA guidelines at baseline visits (2000–2004). Noninvasive vascular assessments were performed within an ancillary study (2012–2017). Tests included carotid–femoral pulse wave velocity, carotid–brachial pulse wave velocity, carotid–radial pulse wave velocity, central pulse pressure, forward pressure wave, and characteristic impedance, as well as brachial artery baseline and hyperemic flow velocities. Linear regression models, adjusted for age, age squared, sex, and heart rate, assessed the associations of LE8 composite and component scores with vascular function.

**Results:**

A higher LE8 score was associated with lower carotid–femoral pulse wave velocity (β = −0.32; 95% CI: –0.42, –0.21; *p* < 0.0001), characteristic impedance (β = −0.57; 95% CI: –0.93, –0.20; *p* = 0.0024), forward pressure wave amplitude (β = −0.21; 95% CI: –0.26, –0.16; *p* < 0.0001), central pulse pressure (β = −0.25; 95% CI: –0.32, –0.19; *p* < 0.0001), and brachial baseline flow velocity (β = −0.013; 95% CI: –0.023, –0.002; *p* = 0.021). Higher LE8 scores were associated with higher brachial hyperemic flow velocity (β = 0.095; 95% CI: 0.035, 0.15; *p* = 0.0018). Blood glucose and blood pressure were the components most significantly associated with vascular function.

**Conclusion:**

Our findings support the concept that a healthy lifestyle is predictive of better vascular function. Future longitudinal studies are warranted to investigate whether improving LE8 scores leads to improved vascular function.

## Background

Cardiovascular disease (CVD) is a significant health burden for the United States, where approximately 800,000 CVD-related deaths occurred in 2021.^1^ Most CVD events are attributable to modifiable lifestyle behaviors, including diet and level of physical activity, which are associated with the lifetime risk of CVD.^2^ In 2010, the American Heart Association (AHA) developed the Life’s Simple 7, which was a framework that defined optimal cardiovascular health with four health behaviors (physical activity, diet, nicotine exposure, and body mass index [BMI]) and three health factors (fasting blood glucose, total cholesterol, and blood pressure).^3^ A higher composite Life’s Simple 7 score is associated with a lower risk of CVD and all-cause mortality.^4^

In 2022, the AHA amended the Life’s Simple 7 framework to include sleep hygiene, thereby establishing the Life’s Essential 8 (LE8).^5^ Additionally, the LE8 introduced a broader and more continuous scoring scheme and modified the criteria for multiple existing health factors to better capture widespread variability in individual health behaviors.^5^ Notably, the new LE8 paradigm has a higher predictive value than its predecessor in predicting the likelihood of adverse CVD events, including myocardial infarction, congestive heart failure, and severe arrythmia, following percutaneous coronary intervention.^6^ A higher LE8 score has also been associated with a decreased risk of incident CVD events in a healthy cohort across multiple age strata.^7^

To date, research has shed light on the predictive power of LE8 for CVD events.^6–8^ Recent findings from the landmark Framingham Heart Study have also suggested an association between LE8 score and measures of macrovascular function, namely aortic stiffness, central pulse pressure (CPP), and carotid–femoral pulse wave velocity (CFPWV).^8^ The deleterious implications of a poor LE8 score are reasonable given that multiple individual components (obesity, actively and formerly smoking, elevated blood pressure, blood glucose, and blood lipid levels) are independently associated with disruptions in vascular function.^9^ This study sought to expand upon the existing literature by assessing the associations of composite LE8 and LE8 components (each factor or behavior individually) with a comprehensive panel of arterial function measures in African Americans, a population at higher baseline risk of CVD and its complications.^10^ Recent research indicates that, even after adjusting for various socioeconomic and neighborhood factors, African American race remains an independent risk factor for CVD.^11^ We examined whether cardiovascular health, assessed by the LE8 score (measured at a mean of 10.9 years, 95% CI: 8.43, 13.35 years earlier), was associated with subsequent measures of vascular function. We hypothesize that a lower composite LE8 score, along with lower LE8 component scores, are associated with subsequent arterial and microvascular dysfunction.

## Methods

Our study was performed in accordance with the Strengthening the Reporting of Observational Studies in Epidemiology (STROBE) reporting guidelines.^12^ Data from this study was obtained from the Jackson Heart Study (JHS), a community-based cohort study assessing CVD risk factors among self-identified African American men and women in Jackson, Mississippi. Through in-person interviews held during baseline visits, social and demographic characteristics, along with medical history data, were collected from study participants. Details for requesting data can be found at the study’s official website: https://www.jacksonheartstudy.org/.

### Participants

**Figure 1.**
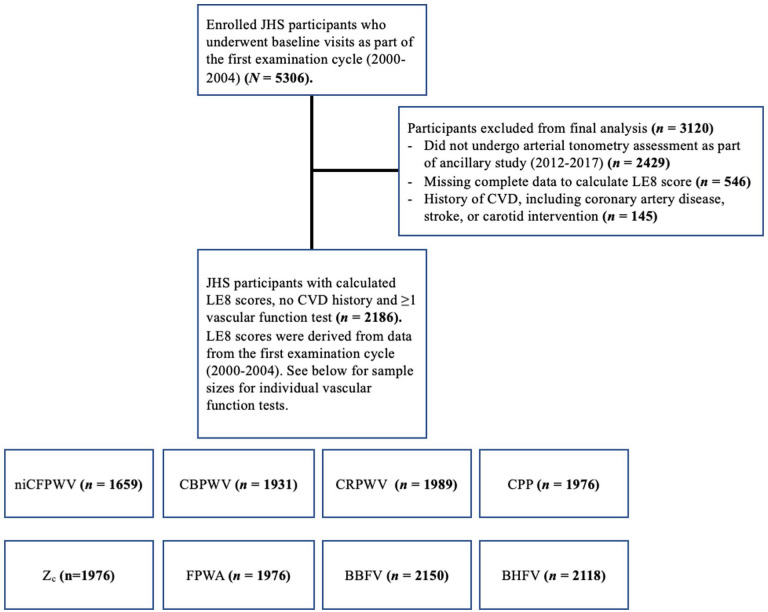
Schematic depiction for inclusion of participants in study analyses. BBFV, brachial baseline flow velocity; BHFV, brachial hyperemic flow velocity; CBPWV, carotid–brachial pulse wave velocity; CPP, central pulse pressure; CRPWV, carotid–radial pulse wave velocity; FPWA, forward pressure wave amplitude; JHS, Jackson Heart Study; LE8, Life’s Essential 8; niCFPWV, negative inverse carotid–femoral pulse wave velocity; Z_c_, characteristic impedance).

The protocol for the JHS has been previously described.^13,14^ This study’s sample involved a subset of JHS participants who underwent arterial tonometry assessment (2012–2017) and had complete data for LE8 score determination at the first examination cycle (2000–2004) (*N* = 2186). Participants who did not have sufficient data for LE8 score determination were excluded. Participants with a history of CVD, including coronary artery disease, stroke, or carotid intervention, were likewise excluded. Figure 1 represents a schematic of the sample selection. Additional participant data pre- and post-stratification by CVD history is provided in Supplemental Tables S1–S3. Written informed consent was procured from all participants in the study, and the study was approved by the institutional review board of the University of Mississippi Medical Center.

### Hemodynamic assessment with arterial tonometry and ultrasound

With participants resting in a supine position, blood pressure was assessed following a 5-minute rest period, as explained previously.^15^ Using simultaneous electrocardiography, tonometry from the brachial, radial, femoral, and carotid arteries was then obtained. Two-dimensional images and pulsed Doppler of the left ventricular outflow tract were obtained using echocardiography. Data were subsequently sent to the core laboratory (Cardiovascular Engineering, Inc., Needham, MA, USA) for blinded analysis. Tonometry waveforms were signal-averaged, and brachial waveforms were utilized to calculate mean arterial pressure per previously described protocols.^15,16^ Integrated mean arterial pressure and diastolic pressures were used to calibrate carotid pressure tracings.^16^ Specific calculations for all vascular function variables were performed as previously described.^17^ CFPWV, carotid–brachial pulse wave velocity, and carotid–radial pulse wave velocity were derived from the ratio of the adjusted transit distance to the pulse transit time difference between the carotid and the femoral, brachial, and radial sites, respectively. An adjustment was made for the carotid–femoral transit distance, as previously described.^18^ CPP was calculated as the difference between carotid systolic and diastolic blood pressures. Forward pressure wave amplitude was defined as the difference between the pressure at the foot of the forward pressure waveform and the pressure at the peak. This was obtained by performing time domain wave separation analysis using flow and central pressure. In the time domain, characteristic impedance was calculated as the ratio of the pressure and flow increase parameters.^19^ These were both measured during the time interval between the onset of flow and the point at which 95% of peak flow was achieved.^19^ Blood density was assumed to be 1.06 g/cm^3^, as previously explained.^20^

### Microvascular function assessment

Using ultrasonography and subsequent image analysis, microvascular function was assessed as previously explained.^21,22^ Brachial artery Doppler flow was acquired at baseline and following a 5-minute ischemic period, which was triggered through inflation of a forearm-positioned cuff. The cuff was positioned immediately distal to the antecubital fold before being inflated to approximately 50 mmHg above systolic blood pressure. Flow was monitored and recorded for a 15-second period following cuff release (until peak flow) by sonographers. Brachial artery images and Doppler flow were assessed with an Acuson S2000 ultrasound system (Siemens Medical Solutions USA, Inc., Mountain View, CA) mounted with a 9L4 transducer with a carrier frequency of 9.0 MHz and an insonation angle of approximately 60°. During acquisition, ultrasound 2D and Doppler audio data were digitized. The data were transferred to the core laboratory (Cardiovascular Engineering) for blinded analyses. Digitized Doppler audio data flows were then analyzed using a semiautomated signal-averaging technique, with the peak flow timing being confirmed via a raw spectral analysis of distinct beats.^23^ Three to five beats (representing the peak flow) were then labeled to produce a signal-averaged spectrum. With electrocardiography (ECG) data serving as a reference point, flow spectra were subsequently signal-averaged. Corrections were made accordingly for the actual insonation angles.

### Life’s Essential 8 (LE8)

The AHA LE8 includes four modifiable health behaviors and four biological factors representing an individual’s cardiovascular health status. Specifically, ideal cardiovascular health involves ⩾ 150 minutes of moderate (or greater) intensity physical activity per week, a Dietary Approaches to Stop Hypertension-style (DASH) diet score ⩾ 95th percentile, a complete lack of any smoking history including electronic cigarette use, approximately 7–9 hours of sleep per night on average, a BMI < 25 kg/m^2^, nonhigh-density lipoprotein cholesterol < 130 mg/dL, a lack of diabetes mellitus history and fasting blood glucose < 100 mg/dL (or glycated hemoglobin [HbA1c] < 5.7%), and a blood pressure < 120/< 80 mmHg.^5^ Per published AHA guidelines, scores on a continuous scale (0–100) were modeled for each component, with an unweighted mean (0–100) representing an individual’s composite LE8 score.^5^ The method by which LE8 scores were derived among JHS study participants by the JHS Coordinating Center is outlined in Supplemental Tables S4 and S5.

### Health behaviors under the LE8 score

Self-reported combustible tobacco use data were obtained from participants and categorized as either current (0 points) or never (100 points). Participants who were former tobacco users or ever exposed to secondhand smoking were scored as outlined in Table S4. For dietary score calculation, the DASH diet score was calculated as previously described.^24^ Components of the dietary score included total fat, saturated fat, fiber, protein, calcium, cholesterol, magnesium, and potassium.^24^ Upon calculating a DASH diet score, the LE8 score was calculated through comparisons with score percentiles from the National Health and Nutrition Examination Survey during the period 1999–2004, with the conversions represented in Table S4. For physical activity, the validated Jackson Heart Study Physical Activity Cohort survey was used to gather data from participants.^25^ A score of 100 was given for weekly physical activity greater than 150 minutes, and scores of 0 were given for 0 minutes per week. Intermediate-range values were scored as described in Table S4. For the sleep component of LE8, participants received a score of 100 points for self-reported sleep durations of 7 to < 9 hours, and 0 points for < 4 hours. Intermediate durations of sleep received scores as outlined in Table S4.

### Health factors under the LE8 score

For nonhigh-density lipoprotein cholesterol, participants received scores of 100 points for values < 130 mg/dL and 0 points for values ⩾ 220 mg/dL. Intermediate-range values were scored as described in Table S5. BMI was calculated with the following formula: weight (kg)/height^2^ (m^2^). Both weight and height were obtained with calibrated devices. BMI was scored as 0 points for participants ⩾ 40 kg/m^2^ and 100 points for < 25 kg/m^2^. Other values were scored as outlined in Table S5. For blood pressure measurements, standard Hawksley random-zero instruments were used to obtain seated blood pressures, 5 minutes apart, at rest. Blood pressure was scored as 0 points for systolic pressure ⩾ 160 mmHg or diastolic pressure ⩾ 100 mmHg and 100 points for < 120/< 80 mmHg, with intermediate values being scored as outlined in Table S5. Diastolic and systolic blood pressure values were calibrated using equations provided by the JHS Coordinating Center, as previously described, and these calibrated values were used in the final LE8 score calculation.^26^ For blood glucose levels, a Vitros 950 or 250 analyzer (Ortho-Clinical Diagnostics, Raritan, NJ, USA) was used to measure fasting levels. Blood glucose was scored as 0 points for ⩾ 10% HbA1c and 100 points for participants without self-reported diabetes mellitus and a HbA1c < 5.7 or fasting blood glucose < 100 mg/dL. Intermediate blood glucose levels were scored as outlined in Table S5.

### Covariates

Covariates were selected a priori as follows: age, age^2^, heart rate, and sex. These parameters were all assessed at the baseline visit. Heart rate was included as it has been shown, through hemodynamic mechanisms, to influence measures of large and peripheral vascular function.^27,28^

### Statistical analysis

The associations of the composite LE8 score and individual LE8 component scores (independent variables) with vascular function measures (dependent variables) were assessed using multivariable-adjusted linear regression models, adjusted for age, age^2^, heart rate, and sex. Continuous variables were entered as native units in all models. To limit heteroscedasticity, CFPWV was first inverted before being multiplied by –1000. Through this mathematical adjustment, higher values corresponded with higher aortic stiffness (hereafter abbreviated as niCFPWV).

A multivariable stepwise analysis was conducted to assess which LE8 components contributed most to the changes in vascular function. Each component score was allowed to be a candidate predictor for each vascular function parameter, with the model ultimately selecting the component scores that were significantly and independently associated with the vascular function result. A *p*-value threshold of *p* < 0.05 was used for variables to be entered into the model, with age, age^2^, heart rate, and sex being included as covariates. All variables were reassessed at each step as new variables were added, and the existing variables with *p*-values of *p* < 0.05 were removed from the model.

For our composite LE8 score model, the threshold *p*-value for significance was likewise *p* < 0.05. To account for multiple comparisons in all statistical models, relations that were statistically significant under a more conservative Bonferroni-adjusted *p* < 0.00625 were noted.^29^ SAS software (version 9.4; SAS Institute, Inc.) was used to conduct the statistical analysis in this study.

## Results

### Baseline characteristics

The characteristics of study participants are presented in Table 1. The LE8 composite score and component score data across study participants are presented in Table 2. The study sample comprised African American adults who were participants in the JHS (*N* = 2186, mean age 57 ± 13 years, 65% women). The mean time interval between baseline LE8 score determination and vascular function test was 10.9 years (95% CI: 8.43, 13.35 years). Of note, the baseline characteristics of our study population closely align with the broader US population. The prevalence of diabetes (JHS: 14.0% vs US: 15.8%), hypertension (JHS: 50.0% vs US: 47.7%), and tobacco smoking history (JHS: 10.0% vs US: 11.6%) are similar.^30–32^ The mean BMI of our study sample (32 ± 6.9 kg/m^2^) is only slightly higher relative to the US population aged 20 years and above: 29.1 kg/m^2^.^33^

**Table 1.** Sample characteristics among included participants from the JHS (*N* = 2186).

Variable	Value
Age, years	57 ± 13
Female sex, *n* (%)	1417 (65)
Body mass index, kg/m^2^	32 ± 6.9
Current smoker, *n* (%)	218 (10)
Ever smoker, *n* (%)	600 (28)
Prevalent hypertension, *n* (%)	1092 (50)
Prevalent diabetes, *n* (%)	302 (14)
Systolic blood pressure, mmHg	126 ± 15
Diastolic blood pressure, mmHg	76 ± 8
Heart rate, beats/minute	66 ± 10
HbA1c, %	5.8 ± 1.0
Low-density lipoprotein cholesterol, mg/dL	128 ± 36
High-density lipoprotein cholesterol, mg/dL	52 ± 14
Triglycerides, mg/dL	104 ± 76
eGFR CKD-EPI score	97 ± 19

All values are represented as mean ± SD or *n* (%).

The eGFR CKD-EPI score is used in the assessment of renal function based on factors including serum creatinine, sex, age, and race.

HbA1c, glycated hemoglobin; JHS, Jackson Heart Study.

**Table 2.** Life’s Essential 8 composite and component scores among included participants from the JHS.

Variable	Value
Life’s Essential 8 composite score	57 ± 13
Diet	26 ± 25
Physical activity	37 ± 40
Tobacco use	78 ± 32
Sleep	73 ± 27
Body mass index	47 ± 32
Blood lipids	60 ± 30
Blood glucose	75 ± 27
Blood pressure	57 ± 32

All values are represented as mean ± SD.

*N* = 2186, mean age 57 ± 13 years, 65% women.

JHS, Jackson Heart Study.

### Associations between composite LE8 score and measures of vascular function

A summary of measures of arterial and microvascular function is presented in Table 3. An overview of derivation methods and physiological significance for each parameter is provided in Table 4. The associations of composite baseline LE8 scores with measures of arterial and microvascular function are presented in Table 5. In models that were adjusted for age, age^2^, heart rate, and sex, a higher composite LE8 score was associated with lower niCFPWV, characteristic impedance, forward pressure wave amplitude, and CPP. Composite LE8 score was not associated with carotid–brachial and carotid–radial pulse wave velocities. Additionally, a higher composite LE8 score was associated with higher brachial hyperemic flow velocity but not with brachial baseline flow velocity. A visual demonstration of the results of this study is provided through Supplemental Figure S1.

**Table 3.** Arterial and microvascular function measures among included participants from the JHS.

Vascular variable	Sample size	Mean ± SD
niCFPWV, ms/m	1659	11.0 ± 4.3
CPP, mmHg	1976	64.8 ± 19.9
FPWA, mmHg	1976	52.7 ± 15.6
Z_c_, (dyne·sec)/cm^5^	1976	266.3 ± 111.0
CBPWV, m/s	1931	9.3 ± 2.3
CRPWV, m/s	1989	9.8 ± 1.8
BBFV, cm/s	2150	5.4 ± 3.2
BHFV, cm/s	2117	47.3 ± 18.8

Values are presented as as mean ± SD.

BBFV, brachial baseline flow velocity; BHFV, brachial hyperemic flow velocity; CBPWV, carotid–brachial pulse wave velocity; CPP, central pulse pressure; CRPWV, carotid–radial pulse wave velocity; FPWA, forward pressure wave amplitude; JHS, Jackson Heart Study; niCFPWV, negative inverse carotid–femoral pulse wave velocity (CFPWV multiplied by –1/1000); Zc, characteristic impedance.

**Table 4.** Summary of vascular function measures, their units, physiological domains, methods of derivation, and physiological significance.

Vascular variable	Physiological domain	Derivation	Significance
niCFPWV (ms/m)	Central, large arterial stiffness	Ratio of transit distance to pulse transit time difference between carotid and femoral arteries	Higher values suggest increased arterial stiffness
CPP (mmHg)	Central, large arterial stiffness	Difference between carotid systolic and diastolic blood pressures	Higher values suggest increased arterial stiffness
FPWA (mmHg)	Central, large arterial stiffness	Difference between the peak and foot of the forward pressure waveform (obtained using tonometry-derived central pressure and Doppler-derived flow as inputs)	Higher values suggest increased arterial stiffness
Z_c_ (dyne·sec/cm^5^)	Central, large arterial stiffness	Ratio of tonometry-derived pressure increase to Doppler-derived flow increase during early systole (from flow onset to 95% of peak flow)	Higher values suggest increased arterial stiffness
CBPWV (m/s)	Peripheral arterial stiffness	Ratio of transit distance to pulse transit time difference between carotid and brachial arteries	Higher values suggest increased arterial stiffness
CRPWV (m/s)	Peripheral arterial stiffness	Ratio of transit distance to pulse transit time difference between carotid and radial arteries	Higher values suggest increased arterial stiffness
BBFV (cm/s)	Microvascular function	Mean Doppler-derived brachial artery flow velocity over multiple cardiac cycles, measured at rest	Lower values suggest diminished microvascular function
BHFV (cm/s)	Microvascular function	Mean Doppler-derived brachial artery flow velocity over multiple cardiac cycles, following release of cuff-induced ischemia	Lower values suggest diminished microvascular function

BBFV, brachial baseline flow velocity; BHFV, brachial hyperemic flow velocity; CBPWV, carotid–brachial pulse wave velocity; CPP, central pulse pressure; CRPWV, carotid–radial pulse wave velocity; FPWA, forward pressure wave amplitude; JHS, Jackson Heart Study; niCFPWV, negative inverse carotid–femoral pulse wave velocity (CFPWV multiplied by –1/1000); Zc, characteristic impedance.

**Table 5.** Associations of composite Life’s Essential 8 (LE8) score with measures of arterial and microvascular function among included participants from the JHS.

Vascular variable	Sample size	Estimated β (95% CI)	*p*
niCFPWV, ms/m	1659	–0.320 (–0.420, –0.210)	< 0.0001^a^
CPP, mmHg	1976	–0.250 (–0.320, –0.190)	< 0.0001^a^
FPWA, mmHg	1976	–0.210 (–0.260, –0.160)	< 0.0001^a^
Z_c_, (dyne·sec)/cm^5^	1976	–0.570 (–0.930, –0.200)	0.0024^a^
CBPWV, m/s	1931	–0.007 (–0.015, 0.002)	0.11
CRPWV, m/s	1989	–0.004 (–0.010, 0.003)	0.24
BBFV, cm/s	2150	–0.013 (–0.023, –0.002)	0.02
BHFV, cm/s	2118	0.095 (0.035, 0.150)	0.0018^a^

Beta regression coefficients (β) represent the estimated change in the outcome variable per 1-unit increase in the composite LE8 score. Models were adjusted for heart rate, age, age^2^, and sex.

a Associations statistically significant under the Bonferroni-adjusted threshold (*p* < 0.00625) for multiple comparisons.

BBFV, brachial baseline flow velocity; BHFV, brachial hyperemic flow velocity; CBPWV, carotid–brachial pulse wave velocity; CPP, central pulse pressure; CRPWV, carotid–radial pulse wave velocity; FPWA, forward pressure wave amplitude; JHS, Jackson Heart Study; niCFPWV, negative inverse carotid–femoral pulse wave velocity (CFPWV multiplied by –1/1000); Zc, characteristic impedance.

### Multivariable stepwise association of components with measures of vascular function

Tables 6 and 7 display a multivariable stepwise analysis, which was conducted to identify LE8 components that contributed to relations between the LE8 composite score and vascular function measures. For the health behaviors, a higher diet score was associated with lower niCFPWV and carotid–brachial pulse wave velocity; a higher physical activity score was associated with lower CPP; a higher smoking exposure score was associated with lower CPP and brachial baseline flow velocity; and sleep was not found to be associated with any of the vascular function measures.

**Table 6.** Multivariable adjusted relations between Life’s Essential 8 (LE8) health behavior component scores with measures of arterial and microvascular function among included participants from the JHS.

Vascular variable	Sample size	Diet Estimated β (95% CI), *p*	Physical activity Estimated β (95% CI), *p*	Smoking Estimated β (95% CI), *p*	Sleep Estimated β (95% CI), *p*
niCFPWV	1659	–0.067 (–0.12, –0.016), 0.010	–	–	–
CPP	1976	–	–0.024 (–0.043, –0.0046), 0.015	–0.035 (–0.060, –0.0099), 0.0062	–
FPWA	1976	–	–	–	–
Z_c_	1976	–	–	–	–
CBPWV	1931	–0.0046 (–0.0087, –0.00057), 0.025	–	–	–
CRPWV	1989	–	–	–	–
BBFV	2150	–	–	–0.008 (–0.012, –0.00379), 0.0002^a^	–
BHFV	2118	–	–	–	–

Data represent individual LE8 health behavior component scores modeled against each vascular function test. All associations that are significant below the *p* < 0.05 threshold are shown.

Beta regression coefficients (β) represent the estimated change in the outcome variable per 1-unit increase in the LE8 component score. Models were adjusted for heart rate, age, age^2^, and sex.

a LE8 component contribution to the association that is statistically significant under the Bonferroni-adjusted threshold (*p* < 0.00625).

BBFV, brachial baseline flow velocity; BHFV, brachial hyperemic flow velocity; CBPWV, carotid–brachial pulse wave velocity; CPP, central pulse pressure; CRPWV, carotid–radial pulse wave velocity; FPWA, forward pressure wave amplitude; JHS, Jackson Heart Study; niCFPWV, negative inverse carotid–femoral pulse wave velocity (CFPWV multiplied by –1/1000); Zc, characteristic impedance.

**Table 7.** Multivariable adjusted relations between Life’s Essential 8 (LE8) health factor component scores with measures of arterial and microvascular function among included participants from the JHS.

Vascular variable	Sample size	BL Estimated β (95% CI), *p*	BG Estimated β (95% CI), *p*	BP Estimated β (95% CI), *p*	BMI Estimated β (95% CI), *p*
niCFPWV	1659	–	–0.11 (–0.16, –0.059), < 0.0001^a^	–0.15 (–0.19, –0.11), < 0.0001^a^	–
CPP	1976	–0.040 (–0.066, –0.014), 0.0027^a^	–0.051 (–0.082, –0.021), 0.001^a^	–0.098 (–0.12, –0.071), < 0.0001^a^	–
FPWA	1976	–0.027 (–0.048, –0.0066), 0.01	–0.067 (–0.091, –0.042), < 0.0001^a^	–0.078 (–0.099, –0.057), < 0.0001^a^	–
Z_c_	1976	–	–0.40 (–0.58, –0.22), < 0.0001^a^	–0.29 (–0.44, –0.14), 0.0001^a^	0.19 (0.042, 0.34), 0.012
CBPWV	1931	–	–	–0.0042 (–0.0075, –0.00088), 0.013	–
CRPWV	1989	–	–	–0.0033 (–0.0059, –0.00063), 0.015	–
BBFV	2150	–	–		–0.0063 (–0.011, –0.002), 0.004^a^
BHFV	2118	–	–	0.039 (0.015, 0.063), 0.0015^a^	–

Data represent individual LE8 health factor component scores modeled against each vascular function test. All associations that are significant below the *p* < 0.05 threshold are shown.

Beta regression coefficients (β) represent the estimated change in the outcome variable per 1-unit increase in LE8 component score. Models were adjusted for heart rate, age, age^2^, and sex.

a LE8 component contribution to the association that is statistically significant under the Bonferroni-adjusted threshold (*p* < 0.00625).

BBFV, brachial baseline flow velocity; BHFV, brachial hyperemic flow velocity; CBPWV, carotid–brachial pulse wave velocity; CPP, central pulse pressure; CRPWV, carotid–radial pulse wave velocity; FPWA, forward pressure wave amplitude; JHS, Jackson Heart Study; niCFPWV, negative inverse carotid–femoral pulse wave velocity (CFPWV multiplied by –1/1000); Zc, characteristic impedance.

For health factors, a higher blood lipids score was associated with lower CPP and forward pressure wave amplitude; a higher blood glucose score was associated with lower niCFPWV, CPP, forward pressure wave amplitude, and characteristic impedance; and a higher blood pressure score was associated with lower niCFPWV, CPP, forward pressure wave amplitude, characteristic impedance, carotid–brachial pulse wave velocity, and carotid–radial pulse wave velocity. A higher blood pressure score was also associated with higher brachial hyperemic flow velocity. Lastly, a higher BMI score was associated with higher characteristic impedance and lower brachial baseline flow velocity.

## Discussion

This study, which leveraged a large community-based sample of African American individuals in Jackson, Mississippi, demonstrates that the AHA’s LE8 health factors and modifiable life behaviors are strongly associated with future trends in arterial and microvascular function. Expanding upon existing research involving central arterial function, the results reveal the LE8 score to be directly associated with measures of microvascular function, namely brachial hyperemic flow velocity.^8^ Our multivariable stepwise model offers insight into the components that contribute most to relations between LE8 score and variability in measures of arterial and microvascular function, with blood pressure and blood glucose being significantly associated with the largest share of vascular function tests. The significant associations in all our models were present even after adjusting for multiple traditional risk factors for poor cardiovascular health.

The vascular function tests utilized in this study represent convenient, noninvasive tools for assessing cardiovascular health. Vascular function is a pertinent parameter to investigate as it reflects the global exposure of individuals to various modifiable lifestyle and CVD risk factors, such as diet and exercise, as well as nonmodifiable risk factors, such as age and genetics. Two major vascular dysfunction phenotypes investigated in this study include diminished microvascular function and large, central arterial stiffening. Microvascular dysfunction is a clinically relevant parameter to assess given that it may often precede macrovascular dysfunction and serve as an independent contributor to CVD.^34^ It has also been independently associated with cardiovascular mortality.^34^ Large artery stiffness shares common cardiometabolic risk factors with microvascular dysfunction, and certain risk factors, including elevated blood pressure, glucose, and lipids, are incorporated into the LE8 score.^5,22^

Although the LE8 score was found to be associated with central macrovascular stiffness measures, such as niCFPWV, it was not found to be associated with peripheral arterial stiffness measures, including carotid–radial and brachial pulse wave velocity. This phenomenon can be explained by physiologic heterogeneity of vessels along the arterial tree, with central arteries tending to be more sensitive than peripheral arteries to various cardiometabolic risk factors.^35^ Of note, central arteries tend to stiffen to a greater degree with factors including age, along with comorbid hypertension and diabetes.^36–39^ From a clinical standpoint, this suggests that, compared to measures of peripheral arterial stiffness, measures of central arterial stiffness may carry better value in reflecting the degree of cardiovascular risk conferred by suboptimal LE8 scores.^36^

Existing research has demonstrated an association between the AHA’s LE8 score with CVD outcomes, with incremental 10-point increases in LE8 score being linked with a significantly lower risk of stroke, myocardial infarction, and heart failure across multiple age strata in adjusted models.^7^ Prior studies have also demonstrated associations between measures of arterial stiffness and future CVD events, with pulse pressure and pulse wave velocity being two parameters shown to be independent predictors of future CVD events.^40,41^ Most recently, research data from the Framingham Heart Study have suggested that poor central arterial function could play a mediating role between LE8 score and all-cause mortality.^8^ Our study broadens the current understanding of the interplay between LE8 and CVD outcomes by offering microvascular dysfunction as a possible mediator, although further longitudinal, outcomes-focused research is warranted to explore this possibility.

Of the LE8 score components, it was found in our study that blood glucose and blood pressure were the most influential drivers of the association between LE8 score and vascular function. The relations of these risk factors to central artery stiffness appear consistent with recent findings from the Framingham Heart Study – although a comparatively weaker significant association between blood glucose and measures of central arterial stiffness had been demonstrated in this prior study.^8^ The results of our study are plausible given the deleterious impact that elevated blood pressure and glucose can have on the vasculature.^42,43^ A possible explanation for the observed closer link between elevated blood pressure and glucose with vascular dysfunction, compared with other LE8 score components, is that elevated blood pressure and glucose are more likely to manifest in cardiovascular complications earlier, often in a subclinical stage of disease.^44–46^ Existing research has also demonstrated that hypertension and diabetes frequently coexist, and that they are often common mediators of other factors, such as obesity and sedentary lifestyle, in triggering CVD.^47,48^

The strengths of this study include the use of a large cohort of African American individuals living in the US who have been extensively phenotyped at a cardio-metabolic level, including extensive vascular function testing and scoring for ideal cardiovascular health metrics. Excluding participants with a CVD history is another strength, as it allows for an assessment of the relations of composite LE8 score and the various component measures with subclinical patterns of vascular dysfunction prior to the manifestation of overt CVD. Our study builds upon the most recent Framingham Heart Study findings, which involved a cohort of participants predominantly of White and European descent.^8^ African Americans have a disproportionately higher risk of experiencing CVD, which manifests earlier in life and may become refractory to conventional treatment.^49^ Previous studies have shown that African Americans have reduced microvascular function, measured as reactive hyperemia index, compared with White individuals.^50^ Through measurement of pulse wave velocity, they were also noted to have higher levels of arterial stiffness.^50^ Strikingly, these differences persisted even in subgroups of individuals completely free of conventional CVD risk factors, such as obesity, hypertension, type 2 diabetes mellitus, and tobacco use.^50^ African race has also been associated with higher fasting blood glucose levels, even among nondiabetic individuals, potentially contributing to a heightened baseline CVD risk.^51^

Our study has multiple limitations. First, the time interval between the participants’ vascular function tests and baseline LE8 score calculations precludes the determination of a pure cross-sectional association, introducing the possibility of confounding factors during the temporal gap. Furthermore, only one set of vascular function tests was performed per study participant following this period, with no further longitudinal follow-up. For the smoking component of the LE8 score, our study methodology interrogated history of tobacco (combustible cigarette) use but did not account for other forms of smoking, such as electronic cigarettes. Although our final analysis did exclude participants with coronary artery disease, stroke, and carotid intervention, these exclusions are certainly not inclusive of every possible form of CVD. Likewise, despite adjusting for multiple cardiovascular risk factors such as age and heart rate, there are likely multiple unaccounted-for confounding variables capable of influencing vascular function. Additionally, because all participants in our study were middle-aged to older African Americans, the results cannot necessarily be generalized to other ethnic or racial groups.

## Conclusions

The AHA’s LE8 was found to be related to future trends in vascular function, including those of the large central arteries and the microvasculature, in this large, community-based cohort study of African American participants. Composite LE8 scores were associated with all vascular function tests aside from those of the peripheral vessels. Specific components of the LE8 score were also found to be associated with vascular function measures, with blood pressure and glucose scores showing significant associations with the largest share of vascular function tests. Sleep, a component introduced to the LE8 from the previous Life’s Simple 7, was not associated with any vascular function test. The results of this study add to the growing body of evidence demonstrating that unhealthy lifestyle behaviors, such as smoking and minimal exercise, and health factors, such as high blood pressure, glucose, and cholesterol, are associated with adverse cardiovascular manifestations. The association between the LE8 score and measures of microvascular function expands upon previous research focused on large central arterial function.^8^ Future longitudinal studies are warranted to further elucidate the complex, multifactorial relationship between the LE8 score and vascular function, and how the LE8 score could potentially predict changes in cardiovascular health status over time.

## Supplemental Material

sj-docx-1-vmj-10.1177_1358863X261444663 – Supplemental material for Relations of Life’s Essential 8 score with arterial and microvascular function: The Jackson Heart Study Supplemental material, sj-docx-1-vmj-10.1177_1358863X261444663 for Relations of Life’s Essential 8 score with arterial and microvascular function: The Jackson Heart Study by Arko S Dhar, William B Hillegass, Leroy L Cooper, Abu Yusuf Ansari, Ramachandran S Vasan, Gary Mitchell and Ervin R Fox in Vascular Medicine

## References

[1] Ritchey MD Wall HK Owens PL , et al. Vital signs: State-level variation in nonfatal and fatal cardiovascular events targeted for prevention by Million Hearts 2022. MMWR Morb Mortal Wkly Rep 2018; 67: 974–982. 30188881 10.15585/mmwr.mm6735a3 PMC6132183

[2] Yusuf S Joseph P Rangarajan S , et al. Modifiable risk factors, cardiovascular disease, and mortality in 155 722 individuals from 21 high-income, middle-income, and low-income countries (PURE): A prospective cohort study. Lancet 2020; 395: 795–808. 31492503 10.1016/S0140-6736(19)32008-2 PMC8006904

[3] Lloyd-Jones DM Hong Y Labarthe D , et al. Defining and setting national goals for cardiovascular health promotion and disease reduction: The American Heart Association’s strategic Impact Goal through 2020 and beyond. Circulation 2010; 121: 586–613. 20089546 10.1161/CIRCULATIONAHA.109.192703

[4] Han L You D Ma W , et al. National trends in American Heart Association revised Life’s Simple 7 metrics associated with risk of mortality among US adults. JAMA Netw Open 2019; 2: e1913131. 10.1001/jamanetworkopen.2019.13131 PMC6804021 31603489

[5] Lloyd-Jones DM Allen NB Anderson CAM , et al. Life’s Essential 8: Updating and enhancing the American Heart Association’s construct of cardiovascular health: A presidential advisory from the American Heart Association. Circulation 2022; 146: e18–e43. 10.1161/CIR.0000000000001078 PMC10503546 35766027

[6] Gao X Ma X Lin P , et al. Predictive value of cardiovascular health score for health outcomes in patients with PCI: Comparison between Life’s Simple 7 and Life’s Essential 8. Int J Environ Res Public Health 2023; 20: 3084. 36833779 10.3390/ijerph20043084 PMC9965286

[7] Ning H Perak AM Siddique J , et al. Association between Life’s Essential 8 cardiovascular health metrics with cardiovascular events in the cardiovascular disease Lifetime Risk Pooling Project. Circ Cardiovasc Qual Outcomes 2024; 17: e010568. 10.1161/CIRCOUTCOMES.123.010568 PMC11209842 38639077

[8] Jha M Dong Z Ruda MM , et al. Associations of cardiovascular health with arterial health in adults free of cardiovascular disease. Am J Prev Cardiol 2025; 23: 101083. 40918934 10.1016/j.ajpc.2025.101083 PMC12409459

[9] Matjuda EN Engwa GA Sewani-Rusike CR , et al. An overview of vascular dysfunction and determinants: The case of children of African ancestry. Front Pediatr 2021; 9: 769589. 34956981 10.3389/fped.2021.769589 PMC8709476

[10] Frierson GM Howard EN DeFina LE , et al. Effect of race and socioeconomic status on cardiovascular risk factor burden: The Cooper Center Longitudinal Study. Ethn Dis 2013; 23: 35–42. 23495620 PMC4266688

[11] Domanski MJ Wu CO Tian X , et al. Association of race with risk of incident cardiovascular disease, coronary heart disease, heart failure, and stroke. JACC Adv 2025; 4: 101811. 40411976 10.1016/j.jacadv.2025.101811 PMC12152612

[12] Von Elm E Altman DG Egger M , et al. Strengthening the Reporting of Observational Studies in Epidemiology (STROBE) statement: Guidelines for reporting observational studies. BMJ 2007; 335: 806–808. 17947786 10.1136/bmj.39335.541782.AD PMC2034723

[13] Carpenter MA Crow R Steffes M , et al. Laboratory, reading center, and coordinating center data management methods in the Jackson Heart Study. Am J Med Sci 2004; 328: 131–144. 15367870 10.1097/00000441-200409000-00001

[14] Taylor HA Jr Wilson JG Jones DW , et al. Toward resolution of cardiovascular health disparities in African Americans: Design and methods of the Jackson Heart Study. Ethn Dis 2005; 15: S6-4–S6-17. 16320381

[15] Mitchell GF Hwang SJ Vasan RS , et al. Arterial stiffness and cardiovascular events: The Framingham Heart Study. Circulation 2010; 121: 505–511. 20083680 10.1161/CIRCULATIONAHA.109.886655 PMC2836717

[16] Kelly R Fitchett D. Noninvasive determination of aortic input impedance and external left ventricular power output: A validation and repeatability study of a new technique. J Am Coll Cardiol 1992; 20: 952–963. 1527307 10.1016/0735-1097(92)90198-v

[17] Cooper LL Musani SK Moore JA , et al. Clinical associations of vascular stiffness, microvascular dysfunction, and prevalent cardiovascular disease in a Black cohort: The Jackson Heart Study. J Am Heart Assoc 2020; 9: e017018. 10.1161/JAHA.120.017018 PMC7726980 32873113

[18] Mitchell GF Wang N Palmisano JN , et al. Hemodynamic correlates of blood pressure across the adult age spectrum: Noninvasive evaluation in the Framingham Heart Study. Circulation 2010; 122: 1379–1386. 20855656 10.1161/CIRCULATIONAHA.109.914507 PMC2981604

[19] Cooper LL Majid S Wang N , et al. Cross-sectional and longitudinal associations of smoking behaviour with central arterial haemodynamic measures: The Framingham Heart Study. Eur J Prev Cardiol. Published online Feb 5, 2025. DOI: 10.1093/eurjpc/zwaf012. PMC12701739 39907713

[20] Mitchell GF Gudnason V Launer LJ , et al. Hemodynamics of increased pulse pressure in older women in the community-based Age, Gene/Environment Susceptibility-Reykjavik Study. Hypertension 2008; 51: 1123–1128. 18259005 10.1161/HYPERTENSIONAHA.107.108175 PMC11106724

[21] Mitchell GF Vita JA Larson MG , et al. Cross-sectional relations of peripheral microvascular function, cardiovascular disease risk factors, and aortic stiffness: The Framingham Heart Study. Circulation 2005; 112: 3722–3728. 16330686 10.1161/CIRCULATIONAHA.105.551168

[22] Cooper LL Musani SK Washington F , et al. Relations of microvascular function, cardiovascular disease risk factors, and aortic stiffness in Blacks: The Jackson Heart Study. J Am Heart Assoc 2018; 7: e009515. 10.1161/JAHA.118.009515 PMC6474961 30371273

[23] Mitchell GF Parise H Vita JA , et al. Local shear stress and brachial artery flow-mediated dilation: The Framingham Heart Study. Hypertension 2004; 44: 134–139. 15249547 10.1161/01.HYP.0000137305.77635.68

[24] Mellen PB Gao SK Vitolins MZ , et al. Deteriorating dietary habits among adults with hypertension: DASH dietary accordance, NHANES 1988–1994 and 1999–2004. Arch Intern Med 2008; 168: 308–314. 18268173 10.1001/archinternmed.2007.119

[25] Smitherman TA Dubbert PM Grothe KB , et al. Validation of the Jackson Heart Study Physical Activity Survey in African Americans. J Phys Act Health 2009; 6(suppl 1): S124–S132. 10.1123/jpah.6.s1.s124 19998858

[26] Seals SR Colantonio LD Tingle JV , et al. Calibration of blood pressure measurements in the Jackson Heart Study. Blood Press Monit 2019; 24: 130–136. 30998553 10.1097/MBP.0000000000000379 PMC7225013

[27] Tan I Spronck B Kiat H , et al. Heart rate dependency of large artery stiffness. Hypertension 2016; 68: 236–242. 27245180 10.1161/HYPERTENSIONAHA.116.07462

[28] Whelton SP Blankstein R Al-Mallah MH , et al. Association of resting heart rate with carotid and aortic arterial stiffness: Multi-ethnic study of atherosclerosis. Hypertension 2013; 62: 477–484. 23836802 10.1161/HYPERTENSIONAHA.113.01605 PMC3838105

[29] Armstrong RA. When to use the Bonferroni correction. Ophthalmic Physiol Opt 2014; 34: 502–508. 24697967 10.1111/opo.12131

[30] Gwira JA Fryar CD Gu Q. Prevalence of total, diagnosed, and undiagnosed diabetes in adults: United States, August 2021–August 2023. NCHS Data Brief 2024; (516): CS354814. 10.15620/cdc/165794 PMC12035661 40085919

[31] Fryar CD Kit B Carroll MD , et al. Hypertension prevalence, awareness, treatment, and control among adults age 18 and older: United States, August 2021–August 2023. NCHS Data Brief 2024; (511): CS354233. 10.15620/cdc/164016 PMC13655856 40085792

[32] VanFrank B Malarcher A Cornelius ME , et al. Adult Smoking Cessation – United States, 2022. MMWR Morb Mortal Wkly Rep 2024; 73: 633–641. 39052529 10.15585/mmwr.mm7329a1 PMC11290909

[33] Fryar CD Kruszon-Moran D Gu Q , et al. Mean body weight, height, waist circumference, and body mass index among adults: United States, 1999–2000 Through 2015–2016. Natl Health Stat Report 2018; (122): 1–16. 30707668

[34] Houben A Martens RJH Stehouwer CDA . Assessing microvascular function in humans from a chronic disease perspective. J Am Soc Nephrol 2017; 28: 3461–3472. 28904002 10.1681/ASN.2017020157 PMC5698072

[35] Yu S McEniery CM. Central versus peripheral artery stiffening and cardiovascular risk. Arterioscler Thromb Vasc Biol 2020; 40: 1028–1033. 32188277 10.1161/ATVBAHA.120.313128

[36] Mitchell GF Guo CY Benjamin EJ , et al. Cross-sectional correlates of increased aortic stiffness in the community: The Framingham Heart Study. Circulation 2007; 115: 2628–2636. 17485578 10.1161/CIRCULATIONAHA.106.667733

[37] Mitchell GF Parise H Benjamin EJ , et al. Changes in arterial stiffness and wave reflection with advancing age in healthy men and women: The Framingham Heart Study. Hypertension 2004; 43: 1239–1245. 15123572 10.1161/01.HYP.0000128420.01881.aa

[38] McEniery CM Yasmin Hall IR , et al. Normal vascular aging: Differential effects on wave reflection and aortic pulse wave velocity: The Anglo-Cardiff Collaborative Trial (ACCT). J Am Coll Cardiol 2005; 46: 1753–1760. 16256881 10.1016/j.jacc.2005.07.037

[39] Utescu MS Couture V Mac-Way F , et al. Determinants of progression of aortic stiffness in hemodialysis patients: A prospective longitudinal study. Hypertension 2013; 62: 154–160. 23648699 10.1161/HYPERTENSIONAHA.113.01200

[40] Mattace-Raso FU van der Cammen TJ Hofman A , et al. Arterial stiffness and risk of coronary heart disease and stroke: The Rotterdam Study. Circulation 2006; 113: 657–663. 16461838 10.1161/CIRCULATIONAHA.105.555235

[41] Cheng W Xu W Luan S , et al. Predictive value of estimated pulse wave velocity with all-cause and cause-specific mortality in the hypertensive population: The National Health and Nutrition Examination Surveys 1999–2014. J Hypertens 2023; 41: 1313–1322. 37260278 10.1097/HJH.0000000000003469

[42] Rask-Madsen C King GL. Vascular complications of diabetes: Mechanisms of injury and protective factors. Cell Metab 2013; 17: 20–33. 23312281 10.1016/j.cmet.2012.11.012 PMC3546345

[43] Fuchs FD Whelton PK. High blood pressure and cardiovascular disease. Hypertension 2020; 75: 285–292. 31865786 10.1161/HYPERTENSIONAHA.119.14240 PMC10243231

[44] Thomas GN Chook P Qiao M , et al. Deleterious impact of ‘high normal’ glucose levels and other metabolic syndrome components on arterial endothelial function and intima-media thickness in apparently healthy Chinese subjects: The CATHAY study. Arterioscler Thromb Vasc Biol 2004; 24: 739–743. 14739120 10.1161/01.ATV.0000118015.26978.07

[45] Bo S Gambino R Gentile L , et al. High-normal blood pressure is associated with a cluster of cardiovascular and metabolic risk factors: A population-based study. J Hypertens 2009; 27: 102–108. 19145777 10.1097/hjh.0b013e328317a7bb

[46] Matsumura T Sankai T Yamagishi K , et al. Impact of major cardiovascular risk factors on the incidence of cardiovascular disease among overweight and non-overweight individuals: The Circulatory Risk in Communities Study (CIRCS). J Atheroscler Thromb 2022; 29: 422–437. 33731540 10.5551/jat.60103 PMC8894112

[47] Wang Z Yang T Fu H. Prevalence of diabetes and hypertension and their interaction effects on cardio-cerebrovascular diseases: A cross-sectional study. BMC Public Health 2021; 21: 1224. 10.1186/s12889-021-11122-y PMC8229421 34172039

[48] Oktay AA Akturk HK Jahangir E. Diabetes mellitus and hypertension: A dual threat. Curr Opin Cardiol 2016; 31: 402–409. 27070651 10.1097/HCO.0000000000000297

[49] Carnethon MR Pu J Howard G , et al. Cardiovascular health in African Americans: A scientific statement from the American Heart Association. Circulation 2017; 136: e393–e423. 10.1161/CIR.0000000000000534 29061565

[50] Morris AA Patel RS Binongo JN , et al. Racial differences in arterial stiffness and microcirculatory function between Black and White Americans. J Am Heart Assoc 2013; 2: e002154. 10.1161/JAHA.112.002154 PMC3647269 23568343

[51] Meigs JB Grant RW Piccolo R , et al. Association of African genetic ancestry with fasting glucose and HbA1c levels in non-diabetic individuals: The Boston Area Community Health (BACH) Prediabetes Study. Diabetologia 2014; 57: 1850–1858. 24942103 10.1007/s00125-014-3301-1 PMC5424892

