# Supplementary material for: Relations of Life’s Essential 8 score with arterial and microvascular function: The Jackson Heart Study
Source: Vasc Med. 2026 Jun 23;31(4):463–73. doi: 10.1177/1358863X261444663 (PMC13451589; doi:10.1177/1358863X261444663)
Supplement: Supplementary file 1 [file sj-docx-1-vmj-10.1177_1358863X261444663.docx]

**SUPPLEMENTAL MATERIAL**

**Association of Life’s Essential 8 Score with Arterial and Microvascular Function: The Jackson Heart Study**

**Authors’ names, academic degrees and affiliations:**

Arko Dhar, BA^1^, William Hillegass MD, PhD^1^, Leroy Cooper, PhD, MPH^2^, Abu Yusuf Ansari^3^, Ramachandran S. Vasan, MD^4^, Gary Mitchell, MD^5^, Ervin Fox, MD^1^

1. University of Mississippi Medical Center, Jackson, Mississippi, USA
2. Vassar College, Poughkeepsie, New York, USA
3. Jackson Heart Study, Coordinating Center, Jackson, Mississippi, USA
4. University of Texas School of Public Health, San Antonio, Texas, USA
5. Cardiovascular Engineering, Inc., Needham, Massachusetts, USA

**Table S1: Baseline characteristics of included versus excluded participants**

| **Variable** | **CVDHx(-) (N=2,186)** | **CVDHx(+) (N=145)** |
| --- | --- | --- |
| Age (years) | 57±13 | 52±13 |
| Female, N (%) | 1,417 (65) | 78 (54) |
| Body mass index, (kg/m^2^) | 32±6.9 | 32±6.7 |
| Current smoker, N (%) | 218 (10%) | 20 (14%) |
| Ever smoker, N (%) | 600 (28%) | 58 (40%) |
| Prevalent hypertension, N (%) | 1092 (50%) | 116 (80%) |
| Prevalent diabetes, N(%) | 302 (14%) | 43 (30%) |
| Systolic blood pressure (mm Hg) | 126±15 | 133±18 |
| Diastolic blood pressure (mm Hg) | 76±8 | 77±9 |
| Heart rate (beats/minute) | 66±10 | 65±11 |
| Hemoglobin A1c (%) | 5.8±1.0 | 6.2±1.3 |
| Low-density lipoprotein cholesterol, (mg/dL) | 128±36 | 116±34 |
| High-density lipoprotein cholesterol, (mg/dL) | 52±14 | 50±15 |
| Triglycerides (mg/dL) | 104±76 | 110±52 |
| eGFRckdepi score | 97±19 | 89±22 |

All values are represented as mean±standard deviation or number (%). EGFRckdepi score is used in the assessment of renal function, based on factors including serum creatinine, sex and age. “CVDHx(-)” denotes participants without cardiovascular disease history, which included history of coronary artery disease, stroke and carotid intervention. “CVDHx(+)” denotes participants with cardiovascular disease history – these participants were excluded from the final analysis.

**Table S2: Life’s Essential 8 composite and component scores of included versus excluded participants**

| **Variable** | **CVDHx(–)**  **(n=2,186)** | **CVDH(+)**  **(n=145)** |
| --- | --- | --- |
| Life’s Essential 8 composite score | 57±13 | 52±13 |
| Diet | 26±25 | 30±28 |
| Physical activity | 37±40 | 33±40 |
| Tobacco use | 78±32 | 72±35 |
| Sleep | 73±27 | 68±31 |
| Body mass index | 47±32 | 46±30 |
| Blood lipids | 60±30 | 62±28 |
| Blood glucose | 75±27 | 75±27 |
| Blood pressure | 57±32 | 43±31 |

All values are mean ± standard deviation**.** “CVDHx(-)” denotes participants without cardiovascular disease history, which included history of coronary artery disease, stroke and carotid intervention. “CVDHx(+)” denotes participants with cardiovascular disease history – these participants were excluded from the final analysis.

**Table S3: Summary of arterial and microvascular function measures of included versus excluded participants**

| **Vascular variable** | **CVDHx(–)**  **(n=2,186)** | **CVDH(+)**  **(n=145)** |
| --- | --- | --- |
| niCFPWV, ms/m (n=1,775) | 11.0±4.3 (n=1,659) | 14.0±5.7 (n=116) |
| Central pulse pressure, mm Hg (n=2,105) | 64.8±19.9 (n=1,976) | 75.0±20.7 (n=129) |
| Forward pressure wave amplitude, mm Hg (n=2,105) | 52.7±15.6 (n=1,976) | 61.1±19.6 (n=129) |
| Characteristic impedance, (dyne·sec)/cm^5^ (n=2,105) | 266.3±111.0 | 210.4±147.8 (n=129) |
| Carotid-brachial pulse wave velocity, m/s (n=2,057) | 9.25±2.27 | 9.9±2.5 (n=126) |
| Carotid-radial pulse wave velocity, m/s (n=2,117) | 9.84±1.83 | 10.3±2.0 (n=128) |
| Baseline brachial flow velocity, cm/s (n=2,294) | 5.44±3.16 | 4.7±3.0 (n=144) |
| Hyperemic brachial flow velocity, cm/s (n=2,261) | 47.3±18.8 | 51.1±13.9 (n=144) |

Mean vascular function test results are denoted as mean ± standard deviation (SD). “ni” denotes the inversion and transformation by – 1/1000 that was used to transform CFPWV. CFPWV indicates carotid-femoral pulse wave velocity. “CVDHx(-)” denotes participants without cardiovascular disease history, which included history of coronary artery disease, stroke and carotid intervention. “CVDHx(+)” denotes participants with cardiovascular disease history – these participants were excluded from the final analysis.

**Table S4: Scoring criteria for Life’s Essential 8 (health behaviors)**

| Domain | CVH metric | JHS Scoring Criteria |
| --- | --- | --- |
| Health behaviors | Diet | Quantile of DASH-style diet adherence  Scoring (population):  Points Quantiles  100 ≥ 95^th^ percentile  80 75^th^-94^th^ percentile  50 50^th^-74^th^ percentile  25 25^th^-49^th^ percentile  0 1^st^-24^th^ percentile |
|  | PA | Minutes of moderate or vigorous leisure physical activity per week  Scoring:  Points Minutes  100 ≥150  90 120-149  80 90-119  60 60-89  40 30-59  20 1-29  0 0 |
|  | Nicotine exposure | Combustible tobacco use; or secondhand smoke exposure  Scoring:  Points Status  100 Never smoker  75 Former smoker, quit ≥5 y  50 Former smoker, quit 1-<5 y  25 Former smoker, quit <1 y  0 Current smoker  Subtract 20 points (unless score is 0) for any close contact with people where they were smoking |
|  | Sleep health | Average hours of sleep per night  Scoring:  Points Level  100 7-<9  90 9-<10  70 6-<7  40 5-<6 or ≥10  20 4-<5  0 <4 |

Method of deriving Life’s Essential 8 score by the Jackson Heart Study Coordinating Center. Abbreviations include CVH (cardiovascular health), PA (physical activity) and DASH (dietary approaches to stop hypertension).

Scores by the Diet: DASH-style diet adherence scores are calculated using the method according to Mellen et al. (2008) in “Deteriorating Dietary Habits Among Adults With Hypertension: Dash Dietary Accordance, NHANES 1988-1994 and 1999-2004.” Population quantiles are based on the distribution of DASH scores reported in Mellen et al. (2008) for the period of 1999-2004, which coincide with the baseline visit of the JHS participants (2000-2004). The DASH scores range from 0 to 9. The DASH scores corresponding to 25%ile, 50%ile, 75%ile and 95%iles in the U.S. population are 1, 2, 3.5 and 5, respectively.

Nicotine: Combustible tobacco includes cigarettes, cigars, cigarillos and pipes. JHS did not directly capture information about living with active indoor smoker in home. Instead, we used the proxy for any close contact with people where they were smoking (“During the past year, about how many hours per week, on the average, were you in close contact with people where they were smoking? For example, at work, your home, in a car, or other close quarters?”).

**Table S5: Scoring criteria for Life’s Essential 8 (health factors)**

| Domain | Cardiovascular health metric | JHS Scoring Criteria |
| --- | --- | --- |
| Health factors | BMI | BMI (kg/m^2^)  Scoring:  Points Level  100 <25  70 25-<30  30 30-<35  15 35-<40  0 ≥40 |
|  | Blood Lipids | Non-HDL cholesterol (mg/dL)  Scoring:  Points Level  100 <130  60 130-159  40 160-189  20 190-219  0 ≥220  Subtract 20 points (unless score is 0) for statin use |
|  | Blood Glucose | FBG (mg/dL) or HbA1c (%)  Scoring:  Points Level  100 No history of diabetes and FBG <100 (or HbA1c<5.7)  60 No diabetes and FBG 100-125 or HbA1c 5.7-6.4 (prediabetes)  40 Diabetes with HbA1c <7  30 Diabetes with HbA1c 7-7.9  20 Diabetes with HbA1c 8-8.9  10 Diabetes with HbA1c 9-9.9  0 Diabetes with HbA1c ≥10 |
|  | BP | Systolic and diastolic BPs (mmHg)  Scoring:  Points Level  100 <120 and <80  75 120-129 and <80  50 130-139 or 80-89  25 140-159 or 90-99  0 ≥160 or ≥100  Subtract 20 points (unless score is 0) for bp meds use |

Method of deriving Life’s Essential 8 score by the Jackson Heart Study Coordinating Center. Abbreviations include CVH (cardiovascular health), BMI (body mass index), HDL (high-density lipoprotein), FBG (fasting blood glucose), and BP (blood pressure)

Blood glucose: JHS did not collect data on prediabetic participants treated with metformin. Note that lower scores should supersede higher scores. For example, a participant with no history of diabetes has FBG=90 and HbA1c=6. The FBG would yield a score of 100, but the HbA1c would yield a score of 60. The final score should be the lower of the two, which is a score of 60.

Blood pressure: note that lower scores should supersede higher scores. For example, a participant has blood pressure of 135/95. The SBP would yield a score of 50, but the DBP would yield a score of 25. The final score should be the lower of the two, which is a score of 25.

**Figure S1: graphical abstract**
